# Molecular Probing of the Stress Activation Volume in Vapor Phase Lubricated Friction

**DOI:** 10.1021/acsami.3c00789

**Published:** 2023-02-24

**Authors:** Chao-Chun Hsu, Liang Peng, Feng-Chun Hsia, Bart Weber, Daniel Bonn, Albert M. Brouwer

**Affiliations:** † van ’t Hoff Institute for Molecular Sciences, University of Amsterdam, Science Park 904, 1098 XH Amsterdam, The Netherlands; ‡ van der Waals-Zeeman Institute, Institute of Physics, University of Amsterdam, Science Park 904, 1098 XH Amsterdam, The Netherlands; § Advanced Research Center for Nanolithography, Science Park 904, 1098 XH Amsterdam, The Netherlands

**Keywords:** fluorescence, molecular rotor, microscopy, vapor phase lubrication, friction

## Abstract

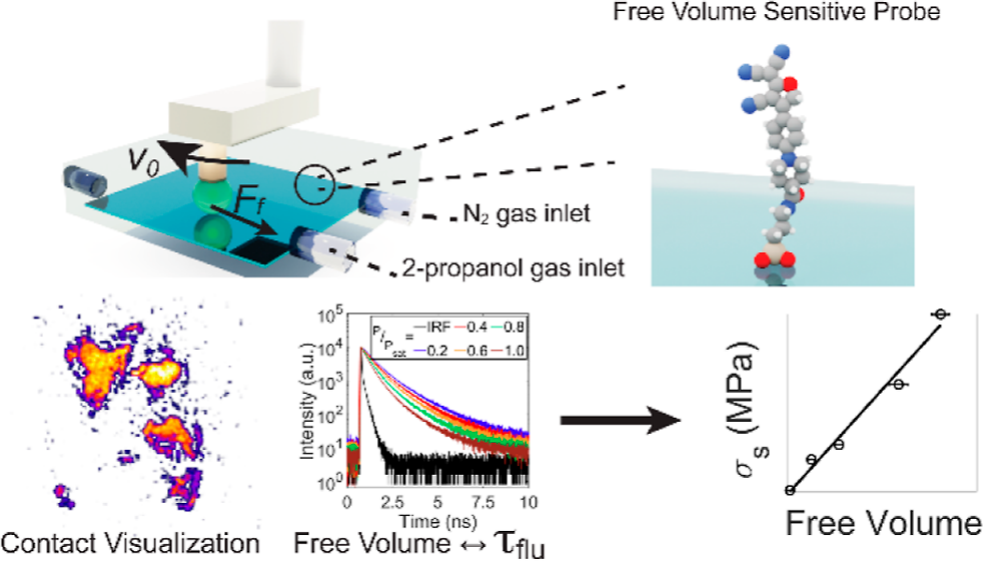

When two solid objects slide over each other, friction results from the interactions between the asperities of the (invariably rough) surfaces. Lubrication happens when viscous lubricants separate the two surfaces and carry the load such that solid-on-solid contacts are avoided. Yet, even small amounts of low-viscosity lubricants can still significantly lower friction through a process called boundary lubrication. Understanding the origin of the boundary lubricating effect is hampered by challenges in measuring the interfacial properties of lubricants directly between the two surfaces. Here, we use rigidochromic fluorescent probe molecules to measure precisely what happens on a molecular scale during vapor-phase boundary lubrication of a polymer bead-on-glass interface. The probe molecules have a longer fluorescence lifetime in a confined environment, which allows one to measure the area of real contact between rough surfaces and infer the shear stress at the lubricated interfaces. The latter is shown to be proportional to the inverse of the local interfacial free volume determined using the measured fluorescence lifetime. The free volume can then be used in an Eyring-type model as the stress activation volume, allowing to collapse the data of stress as a function of sliding velocity and partial pressure of the vapor phase lubricant. This shows directly that as more boundary lubricant is applied, larger clusters of lubricant molecules become involved in the shear process thereby lowering the friction.

## Introduction

When two objects touch, the friction force resists relative motion between the objects at the interface. Friction forces can have unwanted effects such as surface wear, reducing the lifetime of products with moving components, or energy loss due to frictional work. These undesirable side effects of friction can often be significantly reduced by using lubricants: thin films that prevent the two hard surfaces from touching and, consequently, alleviate the shear stress.^1,2^ In (elasto)hydrodynamic lubrication, the lubricant remains in between the two surfaces during frictional sliding because of its local viscosity and layer thickness.^3^ When the lubricant viscosity and sliding velocities are low, the lubricant is squeezed out of the contact, and solid-on-solid friction is observed, generically with high friction coefficients and more wear. However, under some conditions, a few lubricant layers remain at the interface, creating an adsorbed boundary film at the interface, which helps to reduce the friction force. This is known as boundary lubrication; in spite of its importance in many practical situations, the mechanism of boundary lubrication remains elusive.^4^

We address this boundary lubrication problem by studying the lubrication that results from adding only a small amount of lubricating molecules through the vapor phase surrounding a frictional contact. Vapor phase lubrication using alcohols has attracted a lot of attention recently, especially in the field of high precision positioning and microelectromechanical systems.^5−7^ Because alcohols are usually volatile, molecules carried by the vapor easily reach the interface.^8^ The amphipathic nature of alcohols makes them capable of lubricating contacts ranging from hydrophilic–hydrophilic interfaces to hydrophobic–hydrophobic interfaces.^9−11^ It has been suggested that as more alcohol molecules adsorb at solid interfaces the interfacial shear stress decreases.^8,12^ Moreover, surface force apparatus experiments have demonstrated that alcohols are strongly bound to mica surfaces and remain bound even in the presence of contact pressures as large as 100 MPa.^13,14^ Fatty alcohols, such as 1-dodecanol, can even cause superlubricity (extremely low friction) for contact pressures below 1 GPa.^15,16^ Additionally, the alcohol boundary layers can polymerize, especially when subjected to repeated shear, thus preventing fouling of the surfaces.^17−20^ These results all indicate that alcohol vapor phase lubrication is a form of boundary lubrication.

However, the detailed mechanism by which alcohol molecules boundary-lubricate interfaces remains elusive. Displacement of the alcohol molecules during boundary-lubricated sliding was implied by Gates et al.^21^ Briscoe and Evans^22^ used the Eyring model of plastic deformation in solids to describe the effects of boundary layer motion as follows^22,23^
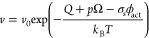
1
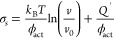
2where σ_*s*_ is the (frictional) shear stress, *k*_B_ is the Boltzmann constant, *T* is the temperature, and *Q*^′^ is the activation energy required to activate the shearing process which equals the sum of the activation energy *Q* and the product of *p* and Ω, which are the normal pressure and the pressure activation volume, respectively. *v* is the sliding speed, *v*_0_ is a characteristic reference velocity, and ϕ_act_ is the stress activation volume. In tribochemical and mechanochemical reactions, the stress activation volume is interpreted as the molecular volume change between the initial state and the transition state so that the reaction rate constant is enhanced by the external force exerted on the reactants. This concept is widely used to explain i.e. tribofilm formation and atomic scale wear.^24−30^ On the other hand, in boundary lubrication, the stress activation volume is interpreted as the volume of boundary layer molecules that are moved at the interface during the thermally activated shear process.^22,24,31−33^ In the remainder of the paper, the stress activation volume is always referred to in the latter meaning. According to eq 2, the effective energy barrier for discrete processes underlying relative motion of the boundary layer decreases with the product of the stress activation volume and the applied shear stress.^24,26,32^ For single-asperity contacts, the dependence of the shear stress on sliding velocity can be accurately captured by the Eyring model.^22,27,28,32,34^ However, for larger multi-contact interfaces, it remains challenging to connect the theoretical description of boundary lubrication to experimental observations due to limitations in our ability to measure the area of real contact and, thus, the shear stress.^4,26^ Moreover, there is no direct method available to locally probe the mobility of boundary layers and connect the activation volume to the free volume of the lubricant. These limitations hamper the translation of fundamental understanding of boundary lubrication into solutions to engineering friction problems.

**Figure 1:**
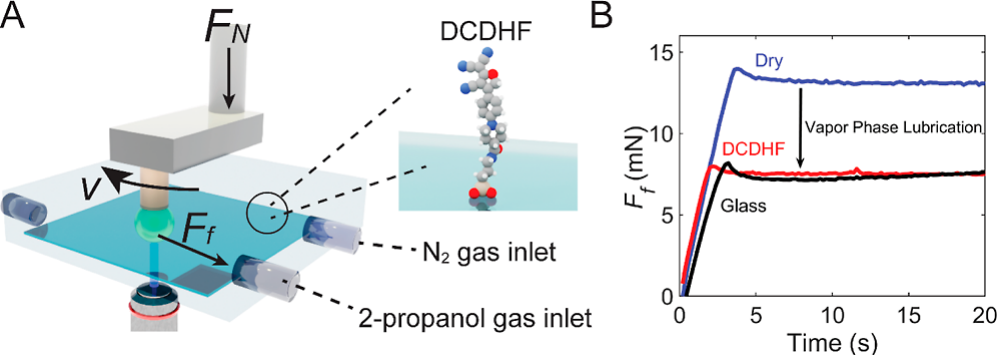
Vapor phase lubricated friction experiments. (A) Experimental setup in which a frictional interface is created by pushing a polymer bead of 3 mm diameter (green) mounted on a rheometer onto a glass surface selectively functionalized with the rigidochromic probe DCDHF (blue). The environment is determined by controlling the flows of dry N_2_ gas and N_2_ gas saturated with 2-propanol vapor. The normal force, *F*_N_, was set to 45 mN and the sliding velocity *v* to 0.25 μm/s. The environment was applied for 40 min before friction measurements were started, to ensure a steady state. (B) Friction force measured in a dry N_2_ environment and in a saturated 2-propanol gas environment at glass coverslips (blue and black). The same friction force was found at DCDHF probe-functionalized coverslips in the presence of 2-propanol (red).

To provide direct insights into both boundary layer mobility and area of real contact, we conducted friction experiments using polypropylene (PP) bead-on-glass interfaces. PP was chosen because it is alcohol resistant.^35^ Controlled friction experiments were conducted by mounting the polymer bead eccentrically to a rheometer plate and lowering and rotating it as indicated in Figure 1A while simultaneously recording the normal and friction forces. To probe the interface mobility, i.e., the free volume of the lubricant at the interface, a confinement-sensitive rigidochromic fluorescent molecule was covalently bonded to the glass surface (Figure 1A). For this purpose, a 2-dicyanomethylene-3-cyano-2,5-dihydrofuran (DCDHF) derivative was selected, which acts as a molecular pressure sensor. In short, this is because the rotation of the bond in the excited state is sensitive to the environmental mobility; in other words, the free volume; the non-radiative decay pathway of the molecule is predominant in a mobile environment, making it non-fluorescent. Upon confinement, the rotation is hampered, thereby promoting radiative-excited state decay: the fluorescence lifetime increases with the degree of confinement as observed both in solution and at solid–solid interfaces.^36−40^ Through fluorescence lifetime imaging microscopy (FLIM), the area of real contact and the local free volume can be visualized in a single measurement.^41^ To investigate vapor phase lubrication of the PP bead-on-glass contact, we performed friction measurements in a small chamber (size 0.5 L) with a controlled atmosphere. The chamber was filled either with dry N_2_ gas (RH <0.8%) or with dry N_2_ gas that was bubbled through two gas washing bottles containing 2-propanol, connected in series, at a flow rate of ∼5 L/min. To obtain various partial pressures of 2-propanol gas in the chamber, saturated 2-propanol gas was mixed with dry N_2_ gas.

Figure 1B shows the results for experiments in dry N_2_ gas versus saturated 2-propanol gas. Immediately after the introduction of saturated 2-propanol gas into an measurement chamber, the friction dropped drastically, reaching a steady state of 60% of the friction measured in dry N_2_ gas. A similar reduction in friction upon vapor phase lubrication with 2-propanol was found for PMMA-on-glass contacts (Figure S1). Figure 1B also shows that the measured vapor phase lubricated friction was not affected by the presence of the DCDHF probe molecules on the glass surface.

**Figure 2:**
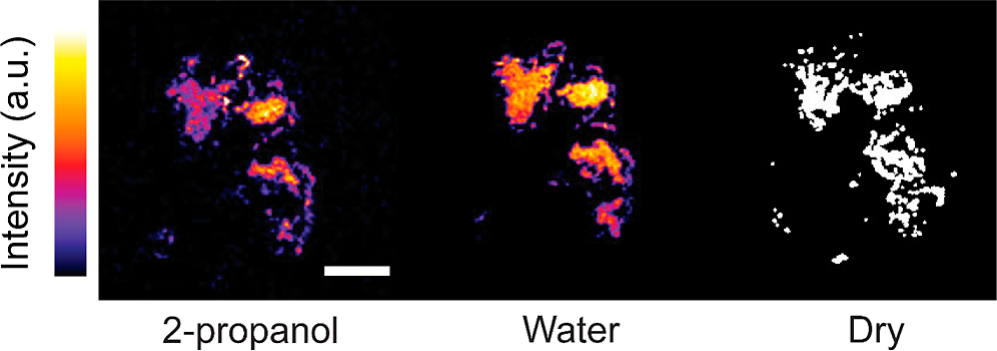
Fluorescence microscopy measurements of the area of real contact at PP-on-glass interfaces lubricated by 2-propanol (left) and water (middle), *F*_N_ = 45 mN. During sliding the area of real contact remained constant (∼4000 μm^2^) and the average contact pressure was approximately 11 MPa. The dry contact area was acquired by a boundary element method (BEM) simulation; the input parameters can be found in the Supporting Information. The average interfacial shear stress was σ_*s*_^IPA^ = 2.3 MPa, σ_*s*_^water^ = 4.1 MPa, and σ_*s*_^dry^ = 3.4 MPa. Scale bar, 50 μm.

To determine if the drop in the friction force upon vapor phase lubrication with 2-propanol is due to a reduction in the contact area and/or interfacial shear strength, we performed sliding experiments with the contact immersed in either liquid 2-propanol or liquid water. During contact and sliding, we used fluorescence confocal microscopy to measure the area of real contact and thus the shear stress. Although diffraction limited fluorescence microscopy has a finite resolution of ∼250 nm, previous studies have demonstrated that for plastic-on-glass interfaces as those studied here, most of the contact structure can be resolved.^37,41^ As the DCDHF molecules at the non-contact area can freely rotate around specific bonds in the molecule, the fluorescence intensity outside the area of real contact is much lower than within the contact. By applying the Otsu thresholding method, we defined the contact area at the lubricated surface, as shown in Figure 2.^30,36−38,41,42^ Comparing those results to the contact area of a dry contact as simulated using the open-source boundary element method (TriboSolver)^43^ based on the elastic-fully plastic half-space approximation (Figure 2),^44^ the contact areas show only minor differences (<5%) between lubricated and dry conditions. Furthermore, we evaluated the topography of the polymer bead before and after the frictional experiment and found no severe wear or deformation (Figure S2). At a similar average contact pressure of ∼11 MPa, the interfacial shear stress was found to vary with lubrication, which we attribute to boundary lubrication. Interestingly, the fluorescence intensity was higher at the contacts with higher shear strength; this result implies that the free volume at the interfaces is related to the interfacial shear stress.

**Figure 3:**
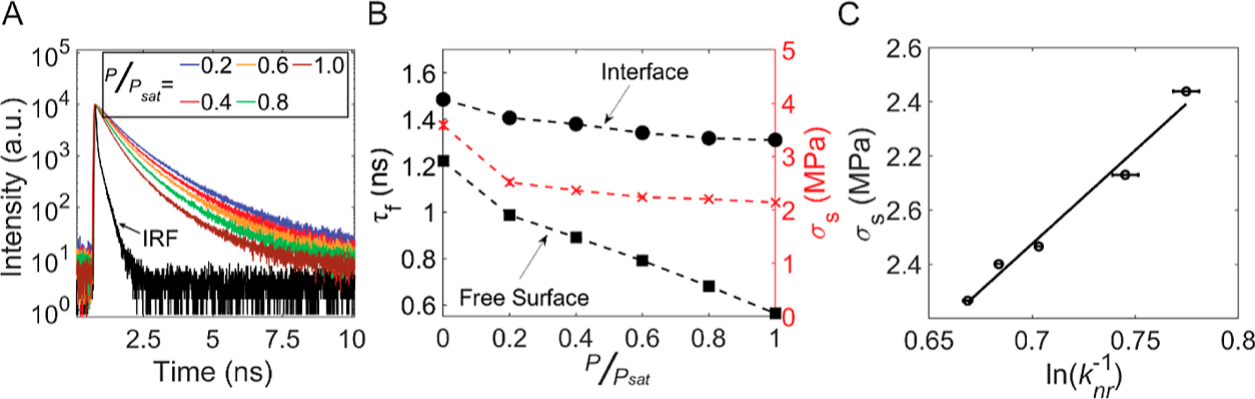
Fluorescence decay of the DCDHF probe molecules and interfacial shear stress of PP-on-glass at various 2-propanol partial pressures. (A) Fluorescence decay curves of the DCDHF probe molecules at different partial pressures *P/P*_sat_ of 2-propanol at the free surface, with IRF designating the instrument response function. Figure S3 shows the corresponding data of the probe molecules confined at the PP-on-glass contacts. (B) Fluorescence lifetimes (τ_*f*_, black symbols) at the free surface (squares) and confined interface (dots) and interfacial shear stress (σ_*s*_, red symbols, obtained as the ratio of friction force and real contact area) as a function of 2-propanol partial pressure. (C) Interfacial shear stress as a function of the logarithm of the inverse of nonradiative fluorescence rate (proportional to the inverse of the free volume) measured at the confined PP bead-on-glass interface. The black line is a linear fit to the data.

To quantitatively probe the free volume at the lubricated interfaces, we measured the fluorescence lifetime of the DCDHF probe molecules both at the free surface and within the contact subject to varying partial pressures of 2-propanol vapor. As shown in Figure 3A, the fluorescence lifetime at both locations decreased as the 2-propanol vapor partial pressure was raised, with the steepest decrease (∼240 ps) occurring on a free surface area when the 2-propanol concentration was increased from 0 to 20%. This indicates that the first layer(s) of 2-propanol already adsorb onto the functionalized surface upon exposure to low partial pressures of 2-propanol gas, thereby increasing the internal mobility of the DCDHF molecules at the surface.^9^ The presence of 2-propanol at the surface continues to grow when the vapor pressure increases, as can be observed from the steadily decreasing fluorescence lifetime. Upon contact with the polymer sphere, the fluorescence lifetime drastically increases at all vapor pressures (black symbols in Figure 3B): the DCDHF molecules experience less free volume due to the imposed contact and pressure.

Next, we evaluated how the vapor pressure of 2-propanol affects the friction force through sliding experiments. The shear stress was found to decrease with increasing partial pressure of 2-propanol vapor (red symbols in Figure 3B), in a comparable fashion as that of the fluorescence lifetime measured within the contact, suggesting a correlation. We probed the free volume at the PP bead-on-glass interface by means of the fluorescence lifetime measured within the contact (see the Supporting Information) using , where *k*_nr_ is the nonradiative decay rate of the rigidochromic molecule and ϕ_free_ is the free volume at the interface.^45^ The measured interfacial shear stress indeed correlates well with the inverse of the free volume: the more free volume the DCDHF molecules experience, the lower the interfacial shear stress. According to eq 2, the shear stress is inversely proportional to the stress activation volume ϕ_act_, which is commonly interpreted as the size of a segment that moves in the unit shear process. Our fluorescence lifetime measurements, therefore, suggest that the DCDHF intramolecular mobility is directly related to this stress activation volume.

**Figure 4:**
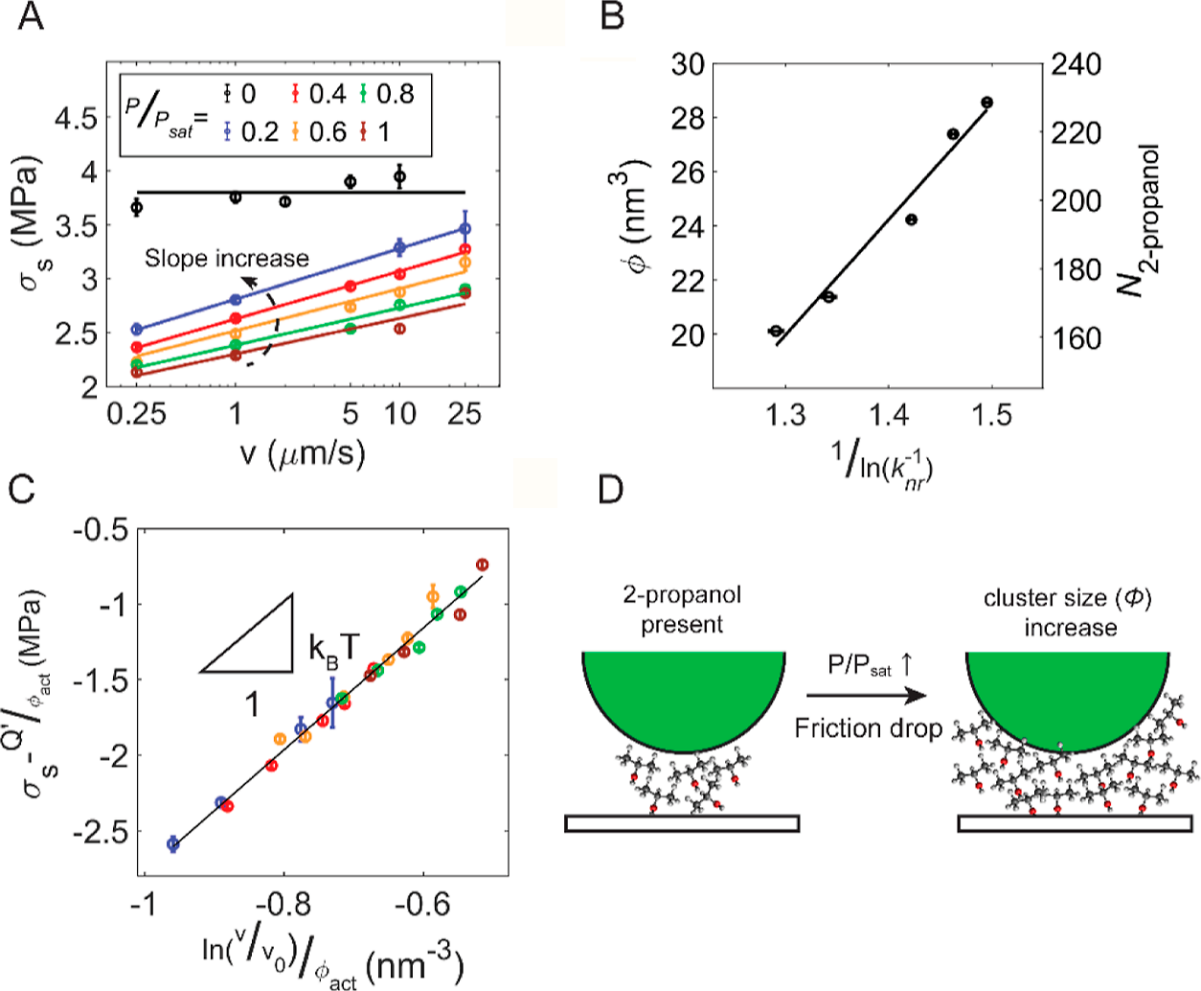
Velocity-dependent interfacial shear stress measurements at varying 2-propanol vapor pressure. (A) Interfacial shear stress as a function of sliding speed (*v*) for different values of *P*/*P*_sat_ between 0 and 1. The solid curves represent linear fits to the data. (B) Stress activation volumes obtained from the data in panel (a) using eq 2 vs. , which is a measure of the free volume obtained from the fluorescence lifetime measurements. The right axis indicates the corresponding number of 2-propanol molecules (N_2-propanol_). (C) Collapse of the friction data from (A) by plotting  versus ln( where *v*_0_ = 60 m/s and *Q*^′^ = 10^–19^ J for all lubrication conditions.^4,22,32,34^ Although *v*_0_ and *Q*^′^ are interchangeable parameters according to eq 2, changing *v*_0_ from 0.1 to 600 m/s only varied the energy barrier, *Q*^′^ by less than 50% (Figure S4). (D) Illustration of the mechanism of the vapor phase lubrication. The 2-propanol molecules will move during the sliding process. As *P*/*P*_sat_ increases, more “mobile” molecules can be sheared in the frictional process, and this further alleviates stress at the interface.

To further corroborate the link between the free volume measured through the DCDHF molecules and the stress activation volume in eq 2, we performed friction experiments at different sliding velocities. In Figure 4A, we plot the interfacial shear stress measured at sliding velocities ranging from 0.25 to 25 μm/s under various 2-propanol vapor pressures. In the absence of vapor phase lubrication, the interfacial shear stress was approximately constant as a function of sliding speed, whereas the interfacial shear stress increased with the logarithm of increasing velocity when the vapor phase lubricant was present. According to eq 2, the slope of the relation between interfacial shear stress and the logarithm of the sliding velocity represents the stress activation volume ϕ, which can thus be extracted from the experimental friction data. In Figure 4B, we plot this stress activation volume against the free volume obtained using the fluorescence lifetime measurements at the confined interface. The two independent measurements are linearly correlated with each other, demonstrating that the free volume probed by the DCDHF molecules and the stress activation volume are strongly connected to each other. To test if eq 2 accurately describes the vapor phase lubricated friction behavior, we chose *v*_0_ to be 60 m/s, a value that was estimated using the size of 2-propanol molecules (6 Å in diameter) and the typical vibration frequency (∼10^11^ Hz) as proposed by Briscoe and Evans*.*^22^ In Figure 4C, we demonstrate that by shifting the sliding velocity with *v*_0_ and with the free volume acquired from Figure 4A and offsetting the interfacial shear stress with , we can collapse all the experimental data onto a single line with the slope equal to *k*_B_*T*. The energy required to activate the shear process, *Q*^′^, was found to be ∼1.0 × 10^–19^ J or 0.6 eV for all lubrication conditions (Figure S4), a typical energy for bonding at the molecular scale.^4,22,32,34^ This energy contains the pressure-induced barrier, i.e., *p*Ω; this suggests that as pressure increases in the contact, the required energy for the shear event will become larger and, thus, hamper the sliding process. A recent study has demonstrated that the contact pressure can hinder tribo–chemical reactions.^46^ Physically, the vapor phase lubricated friction likely involves the local mobility of groups of 2-propanol molecules. As the partial pressure of the vapor phase lubricant is increased, the “mobile” volume also increases, suggesting that larger clusters of 2-propanol become involved in the shear process, as illustrated in Figure 4D.^22^ From our results, the cluster size is ∼20–30 nm^3^: we estimated the corresponding length scale of the cluster as ϕ_act_^1/3^, which gives a few nm, similar to the thickness of the adsorbed 2-propanol film (Figure 4B).^8,47^ These values fall within the cluster volume range of 0.3–350 nm^3^ found in the literature:^22,32,34,48^ the volume is strongly depending on the contacting materials, interface geometry, and size of the contacting asperities.^22,27,28,32,34,49^

## Conclusions

In conclusion, we used rigidochromic fluorescent molecules to probe the free volume at vapor phase-lubricated PP bead-on-glass interfaces. The probe molecules not only enable direct measurement of the free volume at the interface but also allow one to visualize the area of real contact and thus to measure the interfacial stresses that result from externally imposed forces. By comparing the area of real contact and the interfacial shear stress, we found that vapor phase lubrication does not affect the area of real contact but rather the interfacial shear stress. This interfacial shear stress is inversely proportional to the free volume as determined using the fluorescence lifetime measured at the confined interface. Furthermore, we showed that the measured free volume and the stress activation volume in the Eyring model match and that the friction behavior as a function of sliding velocity and vapor pressure are well described by this model. Our results provide new insights into the mechanism of boundary lubrication and contribute to bridging the gap between fundamental single asperity studies and real-world applications involving multi-asperity contacts where lubrication is essential.

## Supporting Information Available

Materials and methods, synthetic procedure to functionalize DCDHF at the glass surface, topography of PP beads before and after sliding experiment, fluorescence decay curve of DCDHF at the contact surface, evalution of energy barrier by varying characteristic velocity, and parameters for BEM simulation (PDF)

## Notes

**Data and materials availability:** data can be found at 10.21942/uva.21884220.
