# Supplementary material for: Molecular Probing of the Stress Activation Volume in Vapor Phase Lubricated Friction
Source: ACS Appl Mater Interfaces. 2023 Feb 24;15(9):12603–8. doi: 10.1021/acsami.3c00789 (PMC9999409; doi:10.1021/acsami.3c00789)
Supplement: Supplementary file 1 — am3c00789_si_001.pdf [file am3c00789_si_001.pdf]

SUPPORTING INFORMATION

Molecular Probing of the Stress Activation Volume in Vapor Phase Lubricated Friction

Chao-Chun Hsu,[1*] Liang Peng,[2] Feng-Chun Hsia,[2,3] Bart Weber,[2,3*] Daniel Bonn, [2] Albert M. Brouwer[1]

[1]van 't Hoff Institute for Molecular Sciences, University of Amsterdam, Science Park 904, 1098 XH Amsterdam, The Netherlands

[2]van der Waals-Zeeman Institute, Institute of Physics, University of Amsterdam, Science Park 904, 1098 XH Amsterdam, The Netherlands

[3]Advanced Research Center for Nanolithography, Science Park 106, 1098 XG Amsterdam, The Netherlands

Email: c.c.hsu@uva.nl (C.C. Hsu), b.weber@uva.nl (B. Weber)

SUPPORTING INFORMATION

Table of Contents

Materials and Methods 3
Supplementary Figures and Tables 4
References 8

Materials and Methods

In Scheme S1 we illustrate the functionalization procedure of 2-dicyanomethylene-3-cyano-2,5-dihydrofuran (DCDHF).[1,2]

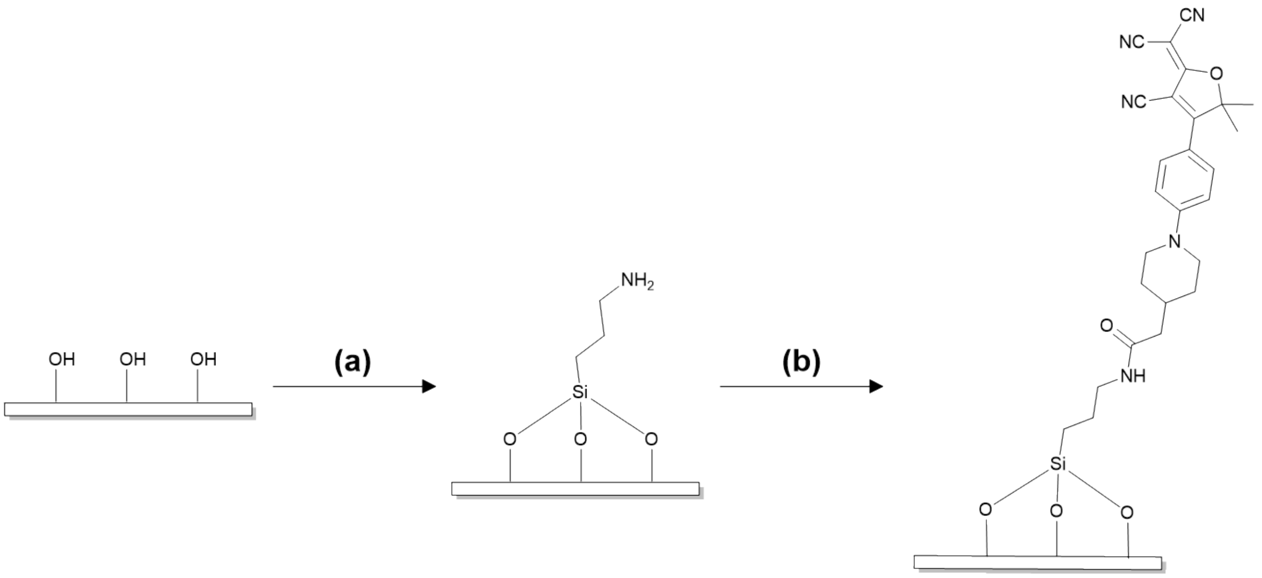

Scheme S1. Synthetic procedure to functionalize DCDHF at the glass surface. (a) APTES, (b) DCDHF, HATU, DIPEA.

Fluorescence lifetimes were measured using a MicroTime 200 confocal microscope (PicoQuant GbmH) with an Olympus IX-71 microscope body and a 100 × 1.4 N.A. objective (UplanSApo, Olympus). Excitation light @488 nm was generated using a Ti:Sapphire Laser (Chameleon Ultra, Coherent; P~2400 mW @ 830 nm; 80 MHz). The laser light (976 nm) passes through second-harmonic generation. The emission from the sample was detected using time-resolved single photon counting (TCSPC) with a PDM Series detector (PicoQuant GmbH). Decay time traces were processed and fitted with SymPhoTime64 with the reconvolution method, and the instrument response function (IRF) was measured by the emission of erythrosine B in saturated potassium iodide solution.[3,4] The resolution of the setup is 4 *ps,* and the full width at maximum of the IRF is *12* ps, i.e., 3 bin-width.

All friction experiments were performed using a rheometer (DSR 301, Anton Paar) mounted on the scanning stage of the microscope. The 2-propanol vapor was brought to the close chamber (~0.5 L) by N_2 gas passing through 2 sequential bubblers containing anhydrous 2-propanol solvent (G1). During the experiment, G1 will be mixed with different ratios of pure N_2 to obtain different amounts of 2-propanol gas in the chamber. Polypropylene (PP) (diameter = 3 mm) and PMMA (d = 1.5 mm) polymer beads (Cospheric, Santa, United States) were used for contact experiment. The contact areas A_r were determined by counting the contact pixels (879 nm/pixel) from the microscopy images applying Otsu thresholding to create a binary image.[5]

The non-radiative decay rate (k_{nr}^{-1}) is calculated using the following equation,

$$\tau = \frac{1}{k_{nr}+k_f} \quad (1)$$

τ is the fluorescence lifetime that we acquired from the TCSPC measurement. k_f Is the radiative decay rate that is ~0.25 ns^{-1} for the DCDHF probe in 2-propanol.[6] Using the relation, we can acquire the non-radiative decay rate, k_{nr}. The k_{nr}^{-1} is proportional to the $exp\left(\frac{1}{\Phi_{free}}\right)$, and we can qualitatively report the free volume at the contact surface.

Supplementary Figures and Tables

Figure S1 demonstrates the friction force measurement on a PMMA-to-glass system. The friction decreases by 50% when the system is blowing with 2-propanol gas (5 L/min).

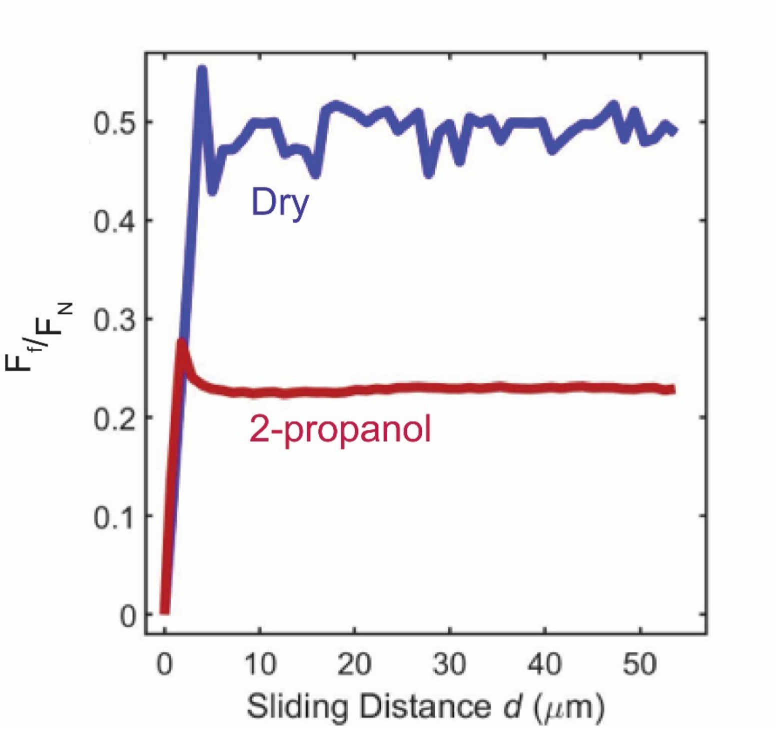

Figure S1 Friction measurement of a PMMA bead sliding on the coverslip without attached dye. The normal force is 100 mN and the sliding speed is 10 μm/s.

Figure S2 shows the topography of the PP bead used in the contact experiment measured using the optical profilometer. The topography was used in the boundary element method to simulate the contact area, and it was compared to the experimental contact area. We found almost identical contact patterns for both methods.

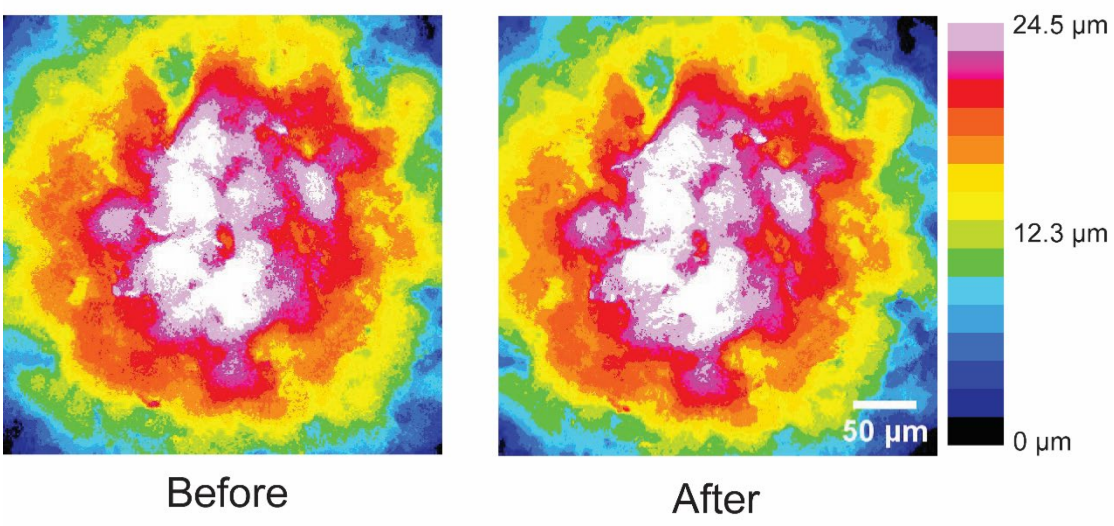

Figure S2 Topography of the PP bead for BEM simulation. (A) Before contact experiment (B) After experiment. The mean square roughness is ~1.7 µm after extracting the global spherical geometry, and we did not observe a severe deformation.

Figure S3 shows the time correlated single photon counting (TCSPC) data of the DCDHF probe inside the contact. On increasing the P/P_{sat} of 2-propanol we observed the lifetime of the probe gradually decreases from 1.47 ns to 1.37 ns.

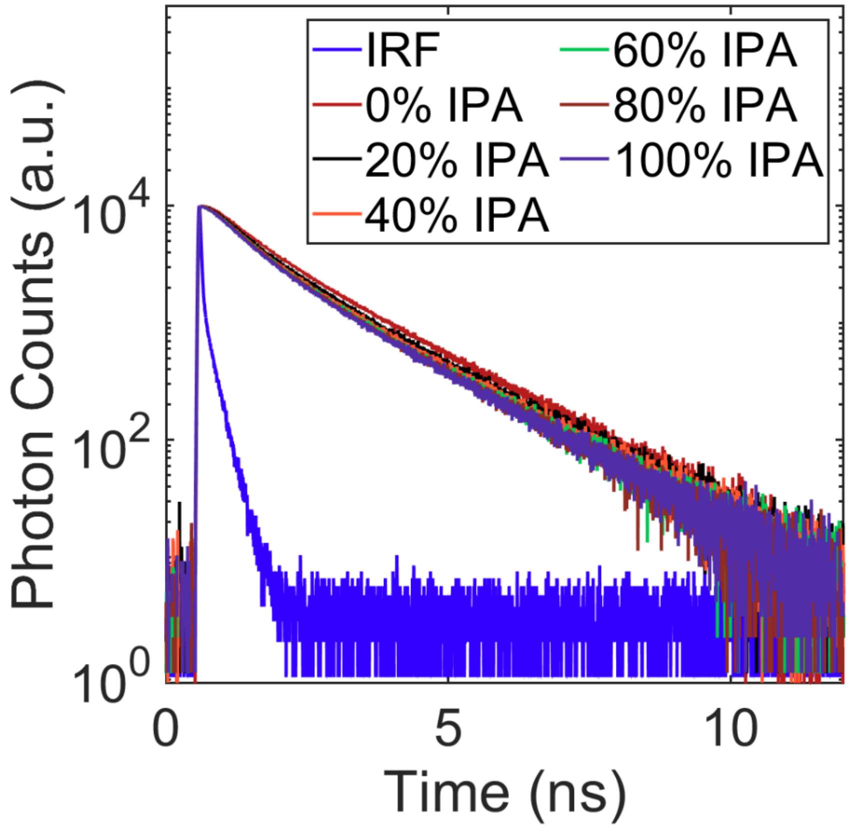

Figure S3 Fluorescence decays of surface-bound DCDHF probe inside the contact with different partial pressures of 2-propanol in the chamber.

In Figure S4, we evaluated the effect of *Q'* by varying v_0 from 0.1 m/s to 600 m/s. In our estimation, we assume $Q' + k_B T ln(v_0) = Const$. Thus, Q' is linearly dependent on $ln(v_0)$. The energy barrier changed <50% with the characteristic velocity change over 3 orders of magnitude. Thus, although we could not precisely acquire the characteristic velocity the *Q'* values still fall in an acceptable range.

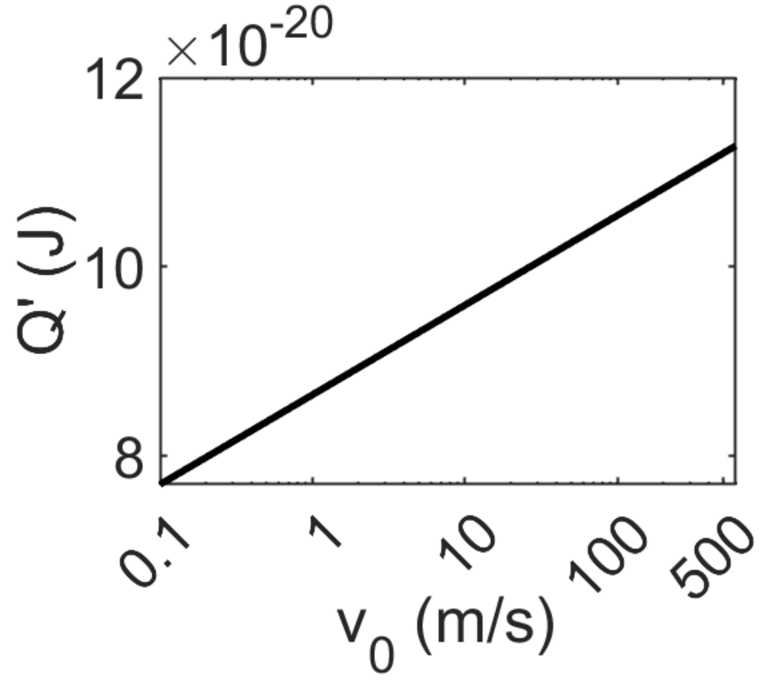

Figure S4 The relation of $\boldsymbol{Q' + k_B T ln(v_0) = Const}$. Q' is linearly dependent on $\boldsymbol{ln(v_0)}$, and Q' has <50% difference with $\boldsymbol{v_0}$ varied from 0.1 to 500 m/s.

SUPPORTING INFORMATION

The input parameters for the BEM simulation can be found in Table S1.

Table S1. Input parameters for BEM simulation.

	E (GPa)	Poisson Ratio	Hardness (GPa)
Glass	70	0.22	5.5
PP	1.3	0.43	0.72

References

(1) Suhina, T.; Weber, B.; Carpentier, C. E.; Lorincz, K.; Schall, P.; Bonn, D.; Brouwer, A. M. Fluorescence Microscopy Visualization of Contacts between Objects. *Angew. Chem. Int. Ed.* **2015**, *54* (12), 3688–3691. https://doi.org/10.1002/anie.201410240.

(2) Weber, B.; Suhina, T.; Junge, T.; Pastewka, L.; Brouwer, A. M.; Bonn, D. Molecular Probes Reveal Deviations from Amontons' Law in Multi-Asperity Frictional Contacts. *Nat. Commun.* **2018**, *9* (1), 888. https://doi.org/10.1038/s41467-018-02981-y.

(3) Szabelski, M.; Ilijev, D.; Sarkar, P.; Luchowski, R.; Gryczynski, Z.; Kapusta, P.; Erdmann, R.; Gryczynski, I. Collisional Quenching of Erythrosine B as a Potential Reference Dye for Impulse Response Function Evaluation. *Appl. Spectrosc.* **2009**, *63* (3), 363–368.

(4) Luchowski, R.; Kapusta, P.; Szabelski, M.; Sarkar, P.; Borejdo, J.; Gryczynski, Z.; Gryczynski, I. Förster Resonance Energy Transfer (FRET)-Based Picosecond Lifetime Reference for Instrument Response Evaluation. *Meas. Sci. Technol.* **2009**, *20* (9), 095601. https://doi.org/10.1088/0957-0233/20/9/095601.

(5) Otsu, N. A Threshold Selection Method from Gray-Level Histograms. *IEEE Trans. Syst. Man. Cybern.* **1979**, *C* (1), 62–66. https://doi.org/10.1109/TSMC.1979.4310076.

(6) Suhina, T.; Amirjalayer, S.; Mennucci, B.; Woutersen, S.; Hilbers, M.; Bonn, D.; Brouwer, A. M. Excited-State Decay Pathways of Molecular Rotors: Twisted Intermediate or Conical Intersection? *J. Phys. Chem. Lett.* **2016**, *7* (21), 4285–4290. https://doi.org/10.1021/acs.jpclett.6b02277.
